# Significance of Textile Properties for the Design of a New Pelvic Implant to Treat Pelvic Organ Prolapse

**DOI:** 10.1007/s00192-025-06370-0

**Published:** 2025-10-09

**Authors:** Amelia Seifalian, Tamara F. Mehdi Bahr Al-Uloom, Nicholas Seifalian, Alex Digesu, Vikram Khullar

**Affiliations:** 1 Department of Urogynaecology, Imperial College London, London, UK; 2 School of Physical and Chemical Sciences, Queen Mary University of London, London, UK; 3 Nanotechnology and Regenerative Medicine Commercialisation Centre, London BioScience Innovation Centre, London, UK

**Keywords:** Polypropylene mesh, Tissue engineering, Pelvic organ prolapse, Textiles, Regenerative medicine, Graphene, Preclinical trials, Biomaterials

## Abstract

**Introduction and hypothesis:**

Pelvic organ prolapse (POP) is a condition that affects millions of women worldwide with grave impact on their mental, social and sexual health. Previously, the polypropylene (PP) mesh was used to augment POP surgery. However, in 2019 the PP mesh was banned across multiple countries due to major complications and women were left with no alternative options. Complications arise, in part, secondary to the textile properties of the PP mesh. Development of a new pelvic implant to overcome previous complications will need to consider all these factors and optimise textile properties to enhance cell behaviour and tissue integration.

**Methods:**

A critical literature review was conducted using PubMed/Medline, Embase, and the Cochrane Library (Wiley). Studies published in the last ten years analysing the textile properties of pelvic implants were included. Eligible papers investigated material composition, fibre type, pore size and porosity, and manufacturing techniques.

**Results:**

PP meshes were associated with stiffness, deformation, and chronic inflammatory responses. Alternative materials such as polyurethane (PU), polyvinylidene fluoride (PVDF), polylactic acid (PLA) demonstrated enhanced elasticity, biocompatibility, and mechanical integrity. Electrospinning and wet spinning allowed finer control over fibre morphology and porosity. Optimal outcomes were observed with pore sizes 1 mm and porosity 50–70%, enabling vascularisation and immune cell infiltration without compromising the implant’s strength.

**Conclusions:**

The textile design of pelvic implants critically determines surgical outcomes and biocompatibility. Future POP implants should utilise elastic, biocompatible materials with optimised pore architecture, fibre geometry, and controlled mechanical properties that replicate the native pelvic floor.

## Introduction

Pelvic organ prolapse (POP) occurs as a result of the descent of pelvic organs bulging down the vagina, secondary to weaknesses of the soft tissue of the pelvic floor. Nearly half of post-menopausal women suffer from this condition—although figures vary due to poor reporting, often overlooked due to its benign nature [1]. Risk factors include vaginal childbirth, age, and obesity, hence there is an increasing prevalence owing to an ageing population and the obesity crises [2]. Current management options include monitoring the condition with pelvic floor exercises, conservative management with vaginal insertion of a replaceable silicone pessary, and invasive surgical procedures of the native tissue or with the use of an adjunct, including synthetic and biologic grafts [3].

Native tissue repair of POP does not provide long-term treatment outcomes with a 33% relapse rate, hence the introduction of an implant to augment the surgery and improve outcomes [4]. Early pelvic mesh implants were adapted from materials used in hernia repair, primarily polypropylene (PP), and were introduced into clinical practice via the FDA’s 510(k) pathway. While initially effective in providing structural support, these implants became associated with high rates of complications, including mesh erosion, chronic pain, and infection [4]. As a result, the FDA mandated their withdrawal from the US market in 2019. Thus, the PP mesh was withdrawn for the treatment of POP by regulatory bodies in the USA, UK, and Canada amongst other countries. In response, research has shifted towards the development of next-generation implants using novel materials and fabrication techniques; however, the majority of these remain at the preclinical stage.

The textile properties of the PP mesh were considered a significant contributor to the long-term complications. PP is a stiff and brittle material found to be prone to breakage and deformation following pelvic implantation [4]. In order for the mesh to successfully integrate with surrounding tissue, the biomechanical properties of the implant need to match that of the pelvic floor [5]. The textile design of the implant is able to manipulate these properties, hence encouraging tissue integration and reducing risk of complication. Textile properties include the material; fibre type and manufacturing technique; pore size and porosity; and fibre thickness. Several PP meshes previously entered the market; each with different features.

The primary aim of this research was to conduct a critical literature review on the material composition, properties of textiles, and design of previous PP pelvic mesh, as well as emerging technology treatment of POP surgery. Finally, we propose a list of desirable textile properties to develop a new pelvic implant with enhanced tissue integration and biocompatibility aiming to mitigate long-term complications.

## Materials and Methods

To complete this comprehensive review, a systematic search was conducted in three major databases PubMed/Medline, Embase and the Cochrane Library (Wiley). The search included studies analysing textile properties of POP implants—published over the past 10 years. Keywords included: “textile properties”, “pelvic mesh”, “pelvic implant”, “POP surgery”, and “biomaterials for pelvic floor repair”. Over 100 articles were identified, of which 15 met the inclusion criteria. Eligible studies were original research in English within the past 10 years on textile properties of pelvic implants; exclusions were non-English papers, abstracts, case reports, reviews, editorials, and studies not analysing textile characteristics.

## Results

Tables 1 and 2 summarised the properties of pelvic implants as reported in the literature over the past 10 years, those that used clinically and emerging technologies, with a focus on the biomaterial fibres utilised and their textile designs. In the subsequent sections, we critically assess the textile designs in terms of their mechanical properties, biocompatibility, tissue integration, and the suitability of each animal model used to evaluate the products.

**Table 1:**
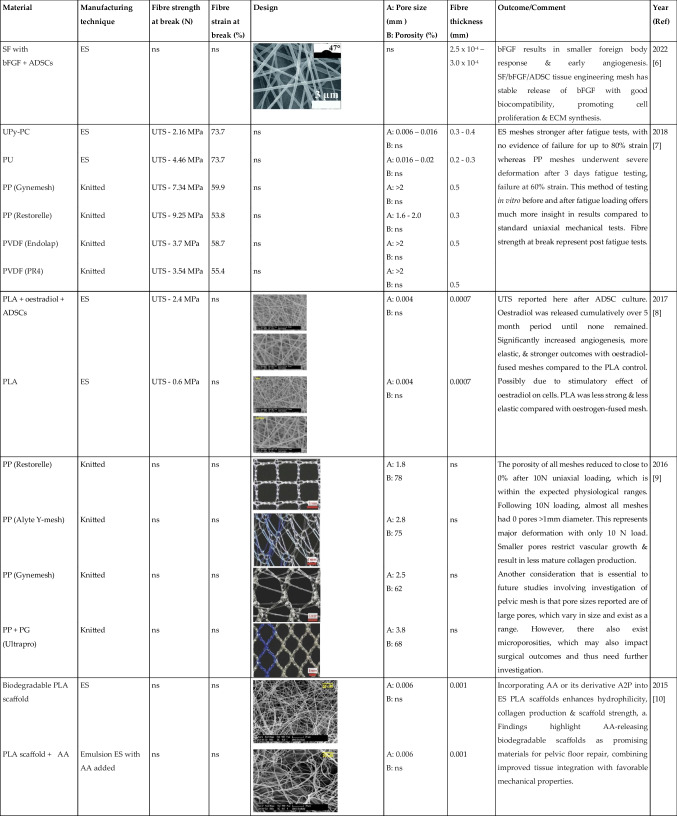
Summary of textile used for manufacturing the pelvic mesh in vitro. Search terms:“Textile properties” and “pelvic mesh” on Pubmed. (Key: A2P, Ascorbate-2-Phosphate; AA, l-ascorbic acid; ADSCs, adipose-derived stem cells; BC, bacterial cellulose; BCCOL, collagen-coated BC; bFGF, basic fibroblast growth factor; ECM, extracellular matrix; ES, electrospun; MF, monofilament; ns, not stated; PLA, polylactic acid; PP, polypropylene; PU, polyurethane; PVDF, polyvinylidene fluroride; SF, silk fibroin; UPy-PC, ureidopyrimidinone-polycarbonate; UTS, ultimate tensile strength; YM, Young’s modulus). Only papers written in the English language were included in the table

**Table 2:**
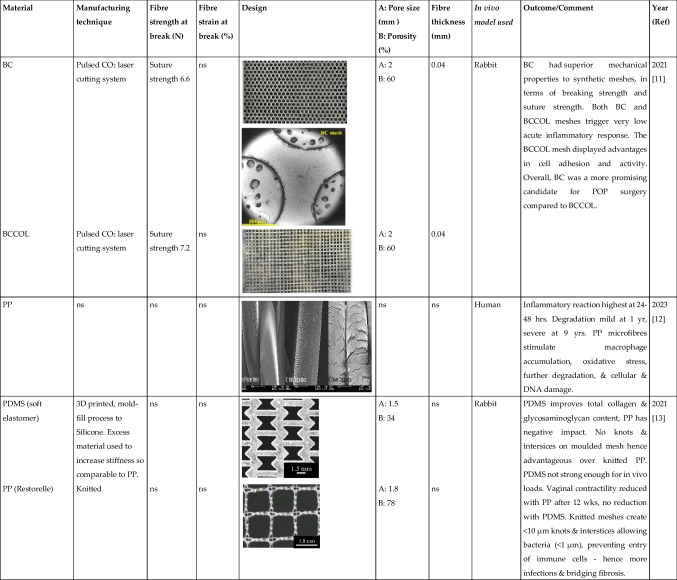

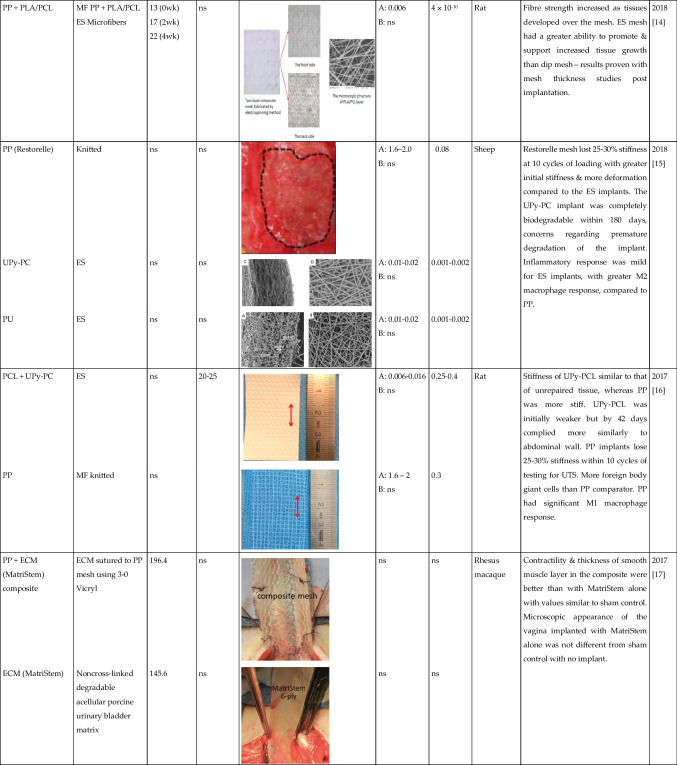

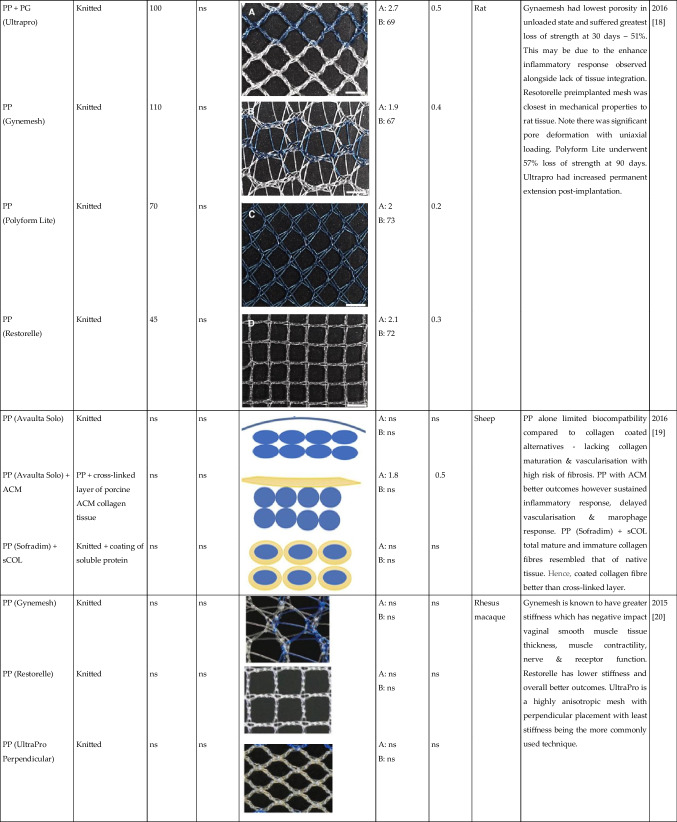
Summary of textile used for manufacturing the pelvic mesh in vivo. Search terms:“Textile properties” and “pelvic mesh” on Pubmed. (Key: ACM, acellular matrix; ECM, extracellular matrix; ES, electrospun; hr, hour; MF, monofilament; ns, not stated; PCL, polycaprolactone; PDMS, polydimethylsiloxane; PLA, polylactic acid; PP, polypropylene; PU, polyurethane; sCOL, solubilised atelocollagen; UPy-PC, ureidopyrimidinone-polycarbonate; UTS, ultimate tensile strength; wk, week; yr, year). Only papers written in the English language were included in the table

### Biomechanical Properties of the Female Pelvic Floor

The female pelvic floor has a significant role in supporting the organs of the pelvis, including the uterus, bladder, and rectum. The main constituent of the pelvic floor is the musculature of the levator ani muscle and the coccygeus. Understanding the biomechanical properties of the pelvic floor are essential for diagnosis of its dysfunction and treatment via the development of a suitable implant for surgical management. It is important that a new implant matches the pelvic floor in terms of stiffness, elasticity and tissue response to dynamic loads, and tensile strength.

Aging, vaginal childbirth, and diminishing hormone production in the post-menopausal state weaken the pelvic floor, leading to POP [2, 21]. Diagnostics for POP remain subjective with first-line being via digital examination. Other methods include ultrasound (US), although with poor resolution for deep tissue and often not useful; magnetic resonance imaging (MRI), however, is considered an expensive and poor use of resources; and finally, manometry for investigating contraction pressure—the most suitable method currently available. Sun et al. performed manometry studies on over 5000 women and confirmed that maximum vaginal contraction pressure was greater in premenopausal nulliparous women compared to both premenopausal parous and postmenopausal women [22], which were the expected outcomes.

Investigation of biomechanical properties of pelvic floor tissue remains complex in living patients due to the invasive nature of accurate testing methods. Vaginal contractility has been used to extrapolate some data on the biomechanical properties of the pelvic floor; however, it is not comparable to standard investigations, including uniaxial testing of mechanical properties [23]. There is, however, more research on the biomechanical properties of cadaveric pelvic tissue, although limitations of this include the poor quality of tissue following degenerative postmortem changes. Nonetheless, mechanical properties have been investigated and data from stress–strain graphs demonstrate pelvic floor high tensile strength—load bearing over 3MPa, whilst remaining elastic and highly ductile—resistant to breakage [24]. The properties described would need to be considered and replicated in the design of a new pelvic implant for POP.

### Summary of Key Textile Design Factors

Across the included studies, several consistent patterns were observed in how textile properties influence pelvic implant performance. Materials such as polyurethane (PU) and polylactic acid (PLA) demonstrated greater elasticity and improved tissue compatibility compared to traditional PP, which was consistently associated with stiffness, deformation under load, and chronic inflammatory responses [12, 15, 17, 25, 26].

Electrospinning (ES) and wet spinning (WS) techniques enabled precise control over fibre diameter and pore architecture. ES produced nanofibrous scaffolds that better mimic the extracellular matrix (ECM), enhancing vascularisation and cell adhesion [7, 10]. However, ES fibres often had pore sizes below the ideal 1 mm threshold, potentially limiting host immune infiltration [9]. WS, used in emerging materials such as graphene-based composites, allowed for more uniform fibre thickness and tunable porosity, addressing mechanical weaknesses observed in ES meshes [27].

Pore sizes greater than 1 mm and porosity levels between 50 and 70% were associated with improved immune cell infiltration, vascularisation, and reduced fibrosis [9, 17, 28]. Denser meshes with low porosity tended to provoke a stronger foreign body response and delayed tissue integration [20]. Fibre thickness also played a role: thinner fibres, particularly those generated by ES, improved biocompatibility and cell interaction but reduced mechanical durability [7, 10]. By contrast, knitted PP meshes maintained strength but caused tissue distortion under load due to their stiffness [18].

Finally, hybrid meshes incorporating biologic coatings such as extracellular matrix (ECM) or collagen, such as MatriStem, resulted in enhanced vascularisation, better smooth muscle layer restoration, and reduced inflammation compared to uncoated PP meshes [17, 19]. These findings underscore the importance of textile design—including pore architecture, fibre type, and surface chemistry—in achieving successful long-term tissue integration of pelvic implants in the treatment of POP.

### Analysis of Textile Characteristics

**Fig. 1:**
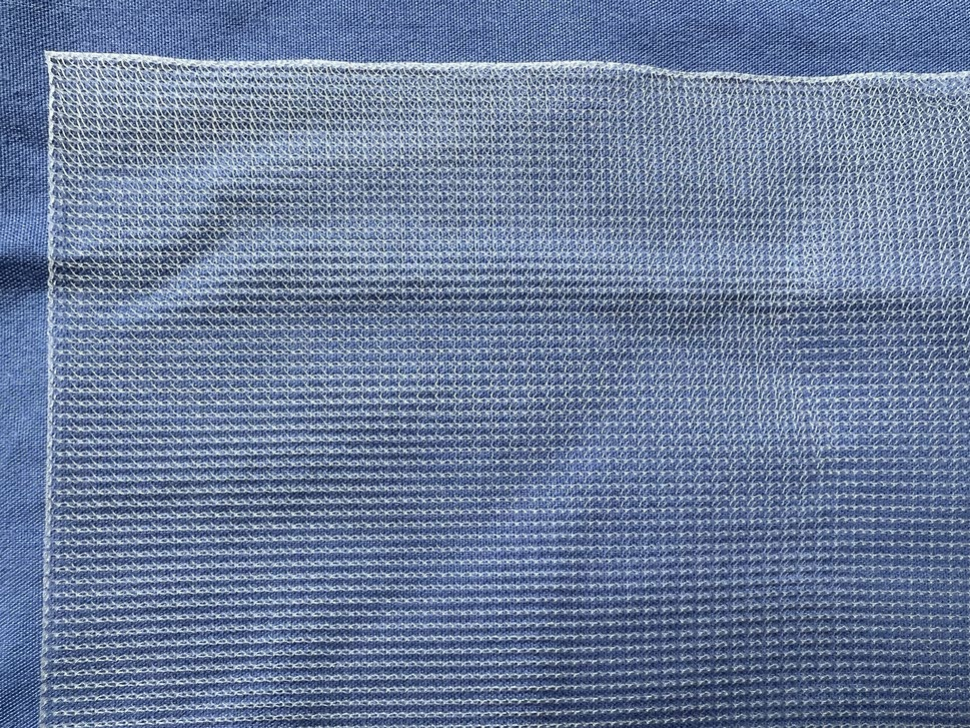
The knitted polypropylene mesh—the most commonly used mesh for pelvic floor repair surgery but now banned across multiple countries due to concerns of long-term complications and poor outcomes

The textile properties of the pelvic meshes were crucial to tissue integration and resulting complications following implantation. The textile properties are impacted by a number of factors: material composition; fibre type and manufacturing technique; pore size and porosity; and fibre thickness. Tables 1 and 2 summarise all pelvic meshes evaluated in vitro and in preclinical animal models, with textile properties highlighted. The most common implants were knitted PP meshes (Fig. 1).

#### Material Composition

The most common synthetic material used to develop pelvic mesh implants has been PP because it is cheap and readily available. PP is a nonabsorbable thermoplastic extruded as a fibre before being knitted as a monofilament or multifilament mesh with its own textile properties [29]. At present, the PP mesh remains banned in multiple countries due to concerns over safety of the material following reports of long-term complications, including chronic pain, chronic infection, and mesh exposure [4].

Polyvinylidene fluoride (PVDF) is a biocompatible and nonabsorbable material that has been used to develop synthetic pelvic mesh. It is a semi-crystalline thermoplastic fluoropolymer. PVDF is more expensive than PP and less readily available in comparison. PVDF has been shown to be more resistant to degradation over time, post implantation [30, 31]. There is evidence to suggest similar cellular response to both PVDF and PP [32]. The main limitations to more widespread use of PVDF include cost—being more expensive than PP—and stiffer when compared to PU, hence why PVDF is not widespread in the POP repair market.

Other materials have also been considered for the development of a pelvic implant, including PU. PU is an organic polymer linked by urethane molecules [33]. Although PP has greater tensile strength, PU is more elastic and therefore better matched to native pelvic tissue [34]. Additionally, PU is able to undergo surface modifications to improve tissue integration via cell adhesion, proliferation, and migration. Research comparing PU and PVDF has shown that PU has better tissue integration outcomes and is better able to maintain its shape and elasticity, compared to PVDF [28, 35]. Although there are concerns regarding durability, early investigations show positive outcomes [7]. PU has not yet been implanted in humans as an implant for POP.

Biodegradable polymers present an alternative to the permanent pelvic implants previously described. Such a material for biodegradable polymers includes PLA. PLA is a thermoplastic derived from organic sources—sugar cane or corn starch [36]. The breakdown product of PLA is nontoxic lactic acid; hence PLA implants are safe for use in humans with minimal complication. However, biodegradable materials risk a lack of mechanical strength when presented as a pelvic implant. The degradation process needs to be at a rate that supports native tissue regeneration and prevents breakdown of mechanical strength [37], hence the current lack of long-term efficacy of biodegradable implants and greater likelihood of POP recurrence.

We have been working on a novel material trade registered as Hastalex® [38] for the development of pelvic membrane as an implant to treat POP. Hastalex is a novel nonbiodegradable material based on functionalised graphene oxide covalently conjugated to the base polymer. It is currently under development for various clinical applications, including heart valves [39], nerve regeneration [40], tendons, and other surgical implants. Its biocompatibility and non-toxicity have been successfully tested [27].

**Fig. 2:**
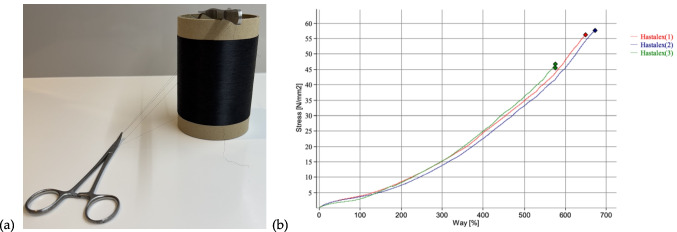
(**a**) Monofilament fibres made from Hastalex®; a graphene-based nanocomposite material manufactured using wet spinning technology. (**b**) Graph depicting the mechanical properties of Hastalex material

Graphene nanomaterials exhibit superior physicochemical properties, combining exceptional strength with elasticity, antibacterial activity, and retention. Research on graphene-based materials has demonstrated their benefits in the field of tissue engineering with enhanced outcomes in regard to cell behaviour and tissue integration, preventing infection with antimicrobial properties, and biocompatibility [41]. We have used WS technology to manufacture mono and multifilament fibres with significantly high tensile strength and strain (Fig. 2a and b). The fibres are currently undergoing development for pelvic membranes for treatment of POP [42].

#### Fibre Type and Manufacturing Technique

Textile properties are influenced by the type of fibre used and manufacturing technique. Fibres used for pelvic mesh can broadly be categorised into two main types: monofilament or multifilament. Monofilament fibres consist of a single strand of material, whereas multifilament fibres are composed of several strands of fibre twisted or braided together. The advantages of monofilament fibres include greater tensile strength and durability, whilst reducing chronic infection due to its continuous surface with fewer interstices [43]. However, the disadvantages of monofilament fibres include increased stiffness and less flexibility. Multifilament fibres, on the other hand, tend to be more elastic—in line with native tissue, but more prone to infection at interstices and are less durable with loss of mechanical strength long-term.

Manufacturing technique for fibre development can be via ES or WS. The fibre developed is then processed into flat meshes via knitting or weaving. The most popular method of developing pelvic mesh for POP surgery is to knit monofilament fibres. Knitting offers better flexibility and durability compared to woven mesh [44]. Knitting is the preferred method of processing versus weaving due to outcomes with greater porosity mesh, hence better tissue integration [4].

The ES process includes the use of a charge to create a jet of liquid droplets of polymer solution to produce nanoscale fibres, which are deposited to form a mesh that mimics the properties of ECM. ES produces fine fibres with implants of greater porosity, hence enhanced vascularisation and tissue integration [45]. The main concern with ES is small pore sizes with dense compacted fibres that impede tissue integration and the immune system, complications of chronic infection [46]. In addition, the implant created is irregular, and it is impossible to obtain the same fibre thickness all the time, hence no quality control or standardisation of resulting implant. Currently, there are no ES meshes on the market for widespread use in humans, and the ES process still requires scale-up.

WS is the process of developing fibres by extruding the polymer solution through spinnerets to a coagulation bath. Interactions between the solvent in the polymer solution and non-solvent in the coagulation bath causes precipitation of polymer that then solidifies to form fibre. The fibres then undergo a drying process to complete development. Polymers suitable for this type of processing include PU, PVDF, Hastalex, and PLA [47]. The process can be customised to produce finer fibres and adjust porosity; however, this method is not yet widespread and requires scaling up [48]. Outcomes are regular with fibre developed having even measurements. The fibre produced is breathable with nanopores that enhance tissue integration post-implantation.

#### Pore Size and Porosity

Pore size and porosity are crucial elements of textile properties that determine surgical outcome and success of tissue integration. The pore size has a significant impact on minimising infection. Pores < 1 mm in diameter result in higher rates of chronic infection post implantation, and this is due to the size allowing entrance and thus growth of microorganisms but too small to allow immune cell infiltration [49]. Hence future implant for POP should have minimum pore size of > 1 mm.

Porosity also impacts tissue integration as it effects the density of the material. Implants with greater porosity are more lightweight and have less of a foreign body impact on native tissue [50]. In addition, greater porosity better allows tissue ingrowth and vascularisation surrounding the implant. Optimum porosity has been defined as in the range of 50 to 70%, balancing for sufficient tissue ingrowth yet maintaining structural integrity [14]. PU has demonstrated better outcomes in regard to maintaining porosity compared to PP and PVDF, thought to be due to having better elasticity properties [28].

#### Fibre Thickness

Fibre thickness largely depends on the method of manufacturing used. There is some evidence that finer fibres enhance biocompatibility and reduce foreign body reactions [44]. However, thicker fibres have better tensile strength and durability at the cost of greater stiffness and reduced compliance with native pelvic tissue. The processes of ES and WS impacts fibre diameter, listed in order of increasing thickness of resultant fibre.

ES produces fine nanoscale fibres which enhance cell adhesion, integration, and proliferation, but with negative impact on foreign body response, tensile strength, and long-term mechanical stability [50]. WS results in micro-to-nanometre range fibres and allows for better control of fibre thickness. However, the process needs optimisation and scale-up for mass production of pelvic membrane for POP surgery.

#### Mechanical Properties

The mechanical properties of the surgical implant depend on the textile properties in conjunction with material used. PP is a stiff and brittle thermoplastic [51]. An implant to treat POP should match the biomechanical properties of native pelvic tissue to prevent complications from mismatch, such as chronic pain and mesh exposure [4]. The properties of materials for POP surgery are commonly investigated by uniaxial mechanical studies; however, this is often not reflective of the stress undergone by the material in vivo. Hence, an in vitro experiment has been designed by Roman et al. performing investigation of the material before and after fatigue to better represent the pelvic tissue condition [7].

The mechanical properties of the material used and design of textile have a major influence on its tissue integration and cell behaviour post implantation. Studies on nonhuman primates have shown that implants with greater stiffness have a negative impact on outcomes [20] due to a mismatch of physicochemical properties of the implant with native pelvic floor tissue. In order for a new implant to succeed, the implant should match as closely as possible the properties of the native pelvic floor; however, this may prove difficult as properties are harder to ascertain in vivo and because of the margin of error when cadaveric structures are used due to postmortem changes of musculature and smooth tissue.

## Discussion

The textile properties of pelvic implants play a critical role in determining surgical outcomes and addressing the complications associated with earlier designs, particularly PP meshes. This study highlights key findings regarding material composition, fibre type, manufacturing techniques, pore size, porosity, and fibre thickness, offering insights into the ideal characteristics for next-generation pelvic implants. The textile properties impact the physicochemical properties of the implant, including its tensile strength and flexibility. This leads to impacts on cell behaviour and tissue integration.

There is some recent evidence suggesting the use of biodegradable synthetic materials, and these may work short term. However, in regard to long-term use there is a higher rate of recurrence as tissue may not regenerate at a rate to match the degradation of the implant [17]. Hence, the defect remains persistent even after the implant biodegrades. PU has greater elasticity and emerges as a promising alternative, with superior outcomes in maintaining mechanical properties and biocompatibility compared to PVDF and PP [28, 35]. However, its use remains experimental, highlighting the need for further preclinical and clinical investigations.

ES is a new method of manufacturing, with reported better outcomes compared to traditional knitted mesh implants [7]. However, this method is still in its early research phases and under development—not yet ready for widespread use in humans. Knitted implants still remain the most common and tradition design for surgical application. ES consists of a jet of nanofibres sprayed onto the collector, thus resulting in a net or web of material fibre with irregular pattern. The implant produced tends to be stronger with better flexibility compared to knitted mesh [16]. The randomised nature of the final product results in a lack of standardisation and thus negatively impacts tissue integration, and hence is not a suitable option for surgical implantation.

WS allows for the formation of fibre from elastomers, such as PU. The WS process allows finer control of the output fibres developed, hence better outcomes tailored to enhance tissue integration of the implant. The final product fibre is regular and standardised and thus provides surgical application [52]. The fibre can be mono- or multi-filament and once produced via the WS method is ready to undergo further processing via weaving or knitting. This process can be used in POP surgery but amongst an array of other tissue engineering applications in which strong, elastic fibres of regular size with enhanced tissue integration properties are required.

Pores larger than 1 mm facilitate immune cell infiltration and vascularisation. Pore shape is also an area to be considered, with less research but a study showing pores of square, hexagon, bow tie, spiral, and triangle to have no change in pore area following uniaxial stretching [53]. Greater porosity has demonstrated dampened foreign body reactions whilst promoting tissue ingrowth [9]. The interplay between porosity and mechanical strength remains a key challenge. While higher porosity benefits tissue compatibility, it can also compromise structural integrity of the implant and impact tensile strength. A new mesh would have to find balance between appropriate porosity for tissue integration whilst maintaining structural strength and integrity as a long-term implant.

All new implants entering the market need to go through rigorous testing to prevent complications that led to the banning of PP mesh across multiple countries. Animal studies should be performed in the sheep model due to similarities to human female pelvic tissue as well as being readily available [54]. Currently, POP remains an unmet clinical need, hence researchers are working on new materials and designs to provide a long-term cure for the condition and help millions of women worldwide.

The successful translation of novel pelvic implants from bench to bedside requires more than favorable preclinical performance. Regulatory approval processes demand robust evidence of safety, biocompatibility, and efficacy, including comprehensive preclinical testing and carefully designed first-in-human trials [55]. National Institute for Health and Care Excellence (NICE) guidance has limited recommendations for commercial pelvic meshes to only under special arrangements, with special consideration informed consent and audit participation. [56]. Ethical considerations are equally critical, including independent ethics board (IRB) oversight, transparent reporting of complications, and fully informed patient consent—particularly in the context of mesh-related litigation and prior device withdrawals [57, 58]. Although a full analysis of these regulatory and ethical frameworks lies beyond the scope of this textile design-focused review, their importance is acknowledged as a necessary bridge between innovation and clinical application.

A key strength of this review lies in its focussed assessment of the design and impact of textile properties—including fibre type, pore size, porosity, and mechanical properties—which are often overlooked in broader reviews of pelvic mesh complications. By evaluating both in vitro and in vivo findings across a range of materials, insight is provided into how textile properties influence tissue integration and host response. Compared to previous reviews that have prioritised surgical techniques or post-market complications, our analysis offers insight for researchers and material scientists involved in the development of the next generation of pelvic implants.

This review is limited as a structured narrative review and not registered as a systematic review with no formal risk-of-bias assessment performed due to the heterogeneity in study designs, models, and outcomes. In addition, while clinical relevance is discussed, most data analysed were preclinical, limiting direct extrapolation to patient outcomes. Despite these constraints, this focused review fills a gap in the literature and supports the development of safer, more effective pelvic implants grounded in textile science, providing direction for future translational research.

## Conclusions

The material composition is essential to the development of pelvic mesh with optimal surgical outcomes, including biocompatibility and tissue integration. PP is unsuitable for the development of a pelvic implant without long-term complications. Emerging materials are under development for a new pelvic implant to augment POP surgery, including polyurethane and graphene-based materials. The ideal pelvic floor implant should combine biocompatibility with biomechanical performance that closely mimics native pelvic tissue.

Key textile properties include large pore size (greater than 1 mm), porosity levels between 50% and 70%, controlled fibre thickness, appropriate elasticity, high tensile strength, and a surface architecture that supports cell adhesion and vascularisation. Materials should elicit minimal inflammatory responses and yet maintain long-term integrity post-implantation. Thermoplastic PP fibres tend to be stiff and brittle, hence lacking integration with native pelvic tissue. There are new extrusion techniques for fibre development, an example of which is ES, but this has a negative impact on mechanical properties and lacks regularity, hence having no standard in terms of fibre and pore sizes. In contrast, WS technology produces fibres that are strong and elastic, and feature nanoporous structures, offering excellent properties for the development of a pelvic membrane. The design of implant textiles significantly influences their integration with surrounding tissue, particularly regarding porosities and mechanical compatibility. Currently, the development of a pelvic implant is considered an unmet clinical need. This represents a multimillion dollar industry, with researchers in both academics and industry, worldwide, striving to develop a clinically viable product.

## Data Availability

The data that support the findings of this study are available on request from the corresponding author, A.S.
